# The Environment Within: Exploring the Role of the Gut Microbiome in Health and Disease

**DOI:** 10.1289/ehp.121-a276

**Published:** 2013-09-01

**Authors:** Lindsey Konkel

**Affiliations:** **Lindsey Konkel** is a Worcester, MA–based journalist who reports on science, health, and the environment. She writes frequently for *Environmental Health News* and *The Daily Climate*.

The human genome codes for approximately 23,000 genes,^^1^^ yet some experts have suggested that the total information coded by the human genome alone is not enough to carry out all of the body’s biological functions.^^2^^ A growing number of studies suggest that part of what determines how the human body functions may be not only our own genes, but also the genes of the trillions of microorganisms that reside on and in our bodies.

The genomes of the bacteria and viruses of the human gut alone are thought to encode 3.3 million genes.^^3^^ “The genetic richness and complexity of the bugs we carry is much richer than our own,” says Jayne Danska, an immunologist at the Hospital for Sick Children Research Institute in Ontario, Canada. “They serve as a buffer and interpreter of our environment. We are chimeric organisms.”

**Figure 1:**
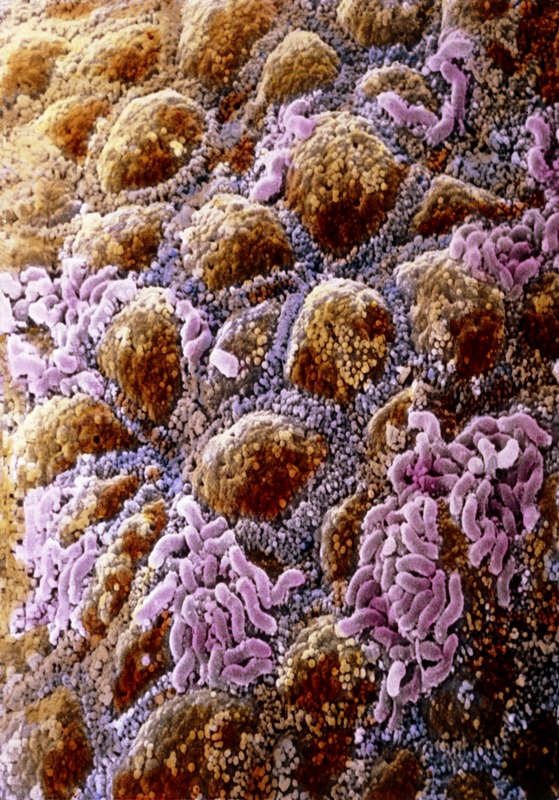
False-color scanning electron micrograph shows the surface of the colon mucosa with pink clusters of rod-shaped bacteria, possibly *Escherichia coli*, attached. The genomes of the bacteria and viruses of the human gut alone are thought to encode 3.3 million genes, which could supplement the human genome in determining how the body functions. © P.M. Motta & F. Carpino/Sapienza University of Rome/Science Source

A role for gut microbes in gastrointestinal function has been well documented since researchers first described differences in the fecal bacteria of people with inflammatory bowel disease.^^4^^ The molecular mechanisms responsible for the gut microbiome’s impact on metabolism and diseases throughout the body remain largely unknown. However, researchers are beginning to decipher how the microorganisms of the human intestinal tract influence biological functions beyond the gut and play a role in immunological, metabolic, and neurological diseases.

## A New Normal

Early research on microbiota focused largely on the commensal bacteria that reside in the human gut. Commensal gut bacteria supply nutrients, help metabolize indigestible compounds, and defend against colonization by nonnative opportunistic pathogens.

But the distinction between “good” microbes that aid health and “bad” pathogenic microbes that cause disease has become blurred in recent years. Researchers have shown that under certain conditions, some types of normal gut bacteria can trigger disease. Sarkis Mazmanian, a microbiologist at the California Institute of Technology, dubbed these elements “pathobionts”; the term “pathogens,” in contrast, refers to opportunistic microbes that are not normally part of the gut microbial community.^^5^^

Disturbances to the microbial equilibrium of the gut may mean that some microbes become overrepresented while others are diminished. “It’s like a garden—you’re less likely to have weeds growing if you have lush vegetation, but without this vegetation the weeds can potentially take over,” Mazmanian says. When the gut moves toward a state of microbial imbalance, normally benign gut microbes may begin to induce inflammation and trigger disease throughout the body, even in the nervous system.

Researchers have long postulated that gut bacteria influence brain function. A century ago, Russian embryologist Elie Metchnikoff surmised that a healthy colonic microbial community could help combat senility and that the friendly bacterial strains found in sour milk and yogurt would increase a person’s longevity.^^6^^^^,^^^^7^^

In 2011 Mazmanian and colleagues reported that changes in gut microbial composition might have far-ranging effects that extend to the brain.^^8^^ They worked with germ-free (“gnotobiotic”) mice, which are born in sterile environments and are not naturally colonized with microbiota.

The researchers found the mice were highly resistant to experimental autoimmune encephalomyelitis (EAE), an animal model for multiple sclerosis, after immunization with central nervous system antigens. These substances that stimulate an immune response and normally induce EAE. However, when some of the mice were intestinally colonized with segmented filamentous bacteria—common commensal inhabitants of the mouse gut—they developed the disease upon being immunized with the central nervous system antigens.^^8^^

Although the study suggested that gut bacteria could affect neurologic inflammation, how that might happen remains unclear. For the most part, Mazmanian says, the microorganisms that colonize the human gut don’t leave the intestine, but the immune cells that contact them do. He explains that, although 70% of the immune cells in the body at any one time can be found in the intestine, they circulate throughout the body, and the microbiota of the gut environment help determine how immune cells will behave elsewhere. He gives an example: “If T-cells, while in the gut, are programmed by the microbiota to have anti-inflammatory properties, then they may suppress inflammation even after they leave the gut.”

Proteins, carbohydrates, and other molecules shed by microbes also leave the gut and may play a role in signaling disease. Studies have shown these bacterial metabolites are pervasive throughout the body—in the lungs,^^9^^ amniotic fluid,^^10^^ and breast milk,^^11^^ all tissues once thought to be free of microbial communities.

Other researchers have suggested a link between the gut–brain axis and neuropsychiatric disorders such as autism, depression, and eating disorders. The gut contains microorganisms that share a structural similarity with the neuropeptides involved in regulating behavior, mood, and emotion—a phenomenon known as molecular mimicry. The body can’t tell the difference between the structure of these mimics and its own cells, so antibodies could end up attacking both, potentially altering the physiology of the gut–brain axis.^^12^^

## The Shifting Microbial Landscape

Changes in microbial colonization of the gastrointestinal tract, a process that starts at birth, have been identified as a major risk factor in the development of food-related autoimmune diseases.^^13^^ The infant gut goes through a series of changes over the first several months of life, especially during the transition from breast milk to solid food. By around 2 years of age, the gut microbial composition of the child more closely resembles that of an adult than that of an infant.^^14^^ Although the microbial composition is thought to remain relatively stable after this point, new research has identified key periods of development for the gut microbial community beyond early childhood, including puberty^^15^^ and lactation.^^11^^

Celiac disease is unique among autoimmune diseases because both the key genetic components and the environmental trigger—gluten—are known.^^16^^ However, fewer than 10% of people with a genetic predisposition to celiac disease develop the condition when exposed to gluten, and most develop the disease years after their first gluten exposure.^^14^^ “Genes and an environmental trigger are necessary but not sufficient for disease development. We knew there had to be a third key element,” says Alessio Fasano, chief of pediatric gastroenterology and nutrition at Mass General Hospital for Children in Boston.

Fasano and colleagues hypothesize the answers lie with the health of the gut microbial ecosystem as a whole. In a small study, they analyzed changes in the microbial communities colonizing the guts of approximately 30 infants with a genetic susceptibility to celiac disease between birth and age 2 years.^^16^^

The gut microbial communities of the predisposed infants matured more slowly and were less stable at 24 months than those of the control infants, who had no known genetic susceptibility to celiac disease. What’s more, genetically susceptible infants who were introduced to gluten at age 6 months were more likely to develop antibodies against gluten than those introduced to gluten at age 12 months.^^16^^ These preliminary findings suggest there may be a critical window of susceptibility to the disease, although more study is needed to explore this possibility.

Celiac disease is one of more than 100 known human autoimmune diseases affecting 5–10% of people worldwide. The incidence of nearly all these diseases is higher among women, and their rising rates and sex specificity suggest an environmental and potentially a hormonal component.^^17^^ “We have known for many years that males are protected from autoimmune disease relative to females. We have not yet found a way to use that information to help women with disease,” Danska says.

Although diet may be the most important environmental factor in determining the functional composition of the gut microbiota, Danska and colleagues recently demonstrated an interaction between sex hormones and the microbiota. Using nonobese diabetic (NOD) mice with a genetic susceptibility to type 1 diabetes, they found that male protection against the disease relative to females was associated with early-life gut microbial colonization. They also found that the composition of the gut microbiota was similar in young males and females but started to diverge between the sexes after puberty.^^15^^

In a germ-free environment, NOD males lost their relative protection against diabetes and had lower levels of testosterone than microbe-colonized males, suggesting a protective interaction between testosterone and gut microbes. When the investigators transplanted microbes from the guts of adult male mice into the guts of young females, the females displayed elevated testosterone levels, changes to their microbiota, and strong protection against type 1 diabetes. Even with these hormonal fluctuations, the female mice that received the male microbiota remained fertile.^^15^^

Although the change in testosterone levels was significant and measurable, Danska calls it modest: “Transfer of the male microbiota to young females did not elevate levels of testosterone anywhere near levels of a normal male, though the metabolic and immune effects were profound.” She says this evidence of interplay between hormones and microbes supports the general idea that “even modest changes in signaling from environmental hormones could have significant impacts on the microbiome.”

The microbial composition of breast milk may also have hormonal determinants.^^11^^ In a small preliminary study of 18 mothers, researchers found differences in the microbiota of breast milk between women who underwent an elective cesarean section and those who gave birth vaginally. Interestingly, the breast milk microbiota of those that underwent a nonelective cesarean section more closely matched the microbial communities of mothers who gave birth vaginally. These differences persisted at 1 and 6 months postpartum.

According to the authors, the findings suggest that milk bacteria are not contaminants but a distinct microbial community, and that hormonal signaling initiated during labor may influence microbial transmission to the milk.^^11^^ Previous studies have shown that babies born vaginally have more diverse microbial communities than babies delivered by cesarean section.^^18^^^^,^^^^19^^

## Assessing the Influence of Environmental Agents

Traditional microbiology culture methods have proved largely unsuccessful in helping to determine the identity and function of the members of the gut microbial community, according to Fasano. “Less than one percent of bacteria that live with us in symbiosis has been cultured,” he says. The identity of the gut microbiota he calls “the dark side of the moon.”

“These organisms live so intimately with each other, each one making a substrate upon which others live. We are just learning how to emulate those conditions outside the gut,” Danska says. She says a few laboratories are making progress culturing consortia of human gut commensals in specialized environments called chemostats.

The burgeoning field of metagenomics, a sequencing approach aimed at describing the genetic richness of whole microbial communities, has allowed researchers to probe the diversity of the body’s microbiota much more deeply than traditional culture techniques.^^20^^^^,^^^^21^^ Classical microbiology is focused on questions such as strain-specific identification—whether a strain is pathogenic, its genetic contents, and any antibiotic resistance parameters. Microbiome analyses, on the other hand, can answer higher-order human health questions—namely, Danska says, “What is the functionality of the consortia of bacteria as a whole?”

Although metagenomics has proven a powerful tool in determining the diversity and metabolic potential of the microbiota, new approaches are needed to determine which microbes are active, which are damaged, and which may respond to a given compound.^^22^^ Some of them metabolize environmental toxicants including polycyclic aromatic hydrocarbons and metals such as arsenic, according to Lisa Chadwick, a program administrator in the National Institute of Environmental Health Sciences (NIEHS) Division of Extramural Research and Training.

Research suggests that short-term exposure to xenobiotics alters microbial physiology, community structure, and gene expression. For instance, studies with antibiotics have found immediate decreases in the stability and diversity of the gut microbiota with only partial recovery up to four years after treatment.^^23^^ And researchers at the University of Miami recently found that in mice exposed orally to polychlorinated biphenyls for two days, the overall abundance of bacteria in the gut was 2.2% lower than in unexposed mice, a statistically significant difference. However, physical exercise appeared to dampen changes to the gut microbiota.^^24^^

These findings notwithstanding, the question of which microorganisms, genes, and pathways are involved in xenobiotic metabolism remains largely unanswered.^^22^^ And the impacts on the microbiota of the thousands of different environmental agents to which the body is exposed each day remain largely unstudied.^^25^^^^,^^^^26^^

“As environmental health scientists, we think a lot about the environment and the role it plays in the developmental origins of disease. Early-life exposures may also change the trajectory of how the microbiome develops and contribute to the development of exposure-related diseases later in life,” says Chadwick. “Now that we are starting to get to know the basic biology of the microbiome, we can start applying that research to environmental health.” Chadwick says the NIEHS will award approximately $2 million in grants in fall 2013 to fund microbiome research projects.

Unraveling the function of the microbiota in disease may offer potential clues to treatment. Researchers on the leading edge of microbial therapy are experimenting with fecal transplantation to help restore healthy microbial communities in patients with inflammatory bowel diseases.^^27^^ Others are hopeful that microbiome studies will lead to therapies for a variety of diseases—immunological, metabolic, and neurological—that have been linked to gut bacteria.

“From my perspective, it’s going to be easier to make and sustain changes to the [microbiota] of individuals than to come up with drugs to alter immune pathways,” says Danska. “I believe that within a handful of years, in countries with high-quality medical care, we will start to see routine administration of well-defined combinations of bacteria to children to prevent autoimmune-mediated diseases.”

However, the personalized nature of such treatments may prove an obstacle that investigators will need to overcome. Mazmanian says, “The same therapy may not work for all people. Specific formulations may have to match the genetics of the patient.”

## A Role for Plant Viruses?

Upwards of 95% of the viral DNA in the human gut may come from plant viruses.^28^ Plant viruses are not known to replicate or cause infection in mammals—they lack the specific receptors necessary to enter human cells and do so. But researchers at the University of Louisville recently detected antibodies to tobacco mosaic virus (TMV), a common pathogen found in tobacco, cucumbers, tomatoes, and peppers, in human serum. Researchers found antibodies in the blood of all 60 healthy male study participants, but smokers had higher levels than nonsmokers, suggesting that cigarettes may be a source of the virus.^29^

In what appears to be a case of molecular mimicry, the researchers found that the anti-TMV antibodies reacted with a human mitochondrial membrane protein that has been associated with an increased risk of Parkinson disease in previous work.^29^ Current study author Robert Friedland, a neurologist at the University of Louisville, hopes these findings may shed light on why smokers have a lower risk of certain autoimmune and neurodegenerative diseases, such as Parkinson disease, Alzheimer disease, and ulcerative colitis.

“Plant viruses are virtually off the radar screen in terms of human health,” says Friedland, who first started investigating a role for plant viruses to see if he could find an agent that could initiate a spontaneous autoimmune response against amyloid ®, a protein component of the brain plaques associated with Alzheimer disease. More than a billion dollars has been spent on developing immunotherapy vaccines for Alzheimer disease aimed at amyloid ®, though clinical trials have proved largely unsuccessful.^30^

Friedland postulates that immune responses generated from dietary exposure to proteins similar to those of amyloid ® might be able to induce antibodies that could influence the progression of Alzheimer disease. In 2008 Friedland and colleagues found that antibodies to potato virus Y bind the amyloid ® peptide.^31^
